# High rates of health care utilization in pediatric multiple sclerosis: A Canadian population-based study

**DOI:** 10.1371/journal.pone.0218215

**Published:** 2019-06-11

**Authors:** Ruth Ann Marrie, Julia O’Mahony, Colleen J. Maxwell, Vicki Ling, E. Ann Yeh, Douglas L. Arnold, Amit Bar-Or, Brenda Banwell, for the Canadian Pediatric Demyelinating Disease Network

**Affiliations:** 1 Department of Internal Medicine, Max Rady College of Medicine, Rady Faculty of Health Sciences, University of Manitoba, Winnipeg, Manitoba, Canada; 2 Department of Community Health Sciences, Max Rady College of Medicine, Rady Faculty of Health Sciences, University of Manitoba, Winnipeg, Manitoba, Canada; 3 Institute of Health Policy, Management and Evaluation, University of Toronto, Toronto, Ontario, Canada; 4 Division of Neurology, The Hospital for Sick Children, Toronto, Ontario, Canada; 5 School of Pharmacy, University of Waterloo, Waterloo, Ontario, Canada; 6 School of Public Health and Health Systems, University of Waterloo, Waterloo, Ontario, Canada; 7 ICES, Toronto, Ontario, Canada; 8 Department of Pediatrics, Division of Neurology, University of Toronto; Toronto, Ontario, Canada; 9 Neurosciences and Mental Health, SickKids Research Institute, Toronto, Ontario, Canada; 10 Montreal Neurological Institute, McGill University, Montreal, Quebec, Canada; 11 Center for Neuroinflammation and Experimental Therapeutics, Perelman School of Medicine, University of Pennsylvania, Philadelphia, Pennsylvania, United States of America; 12 Department of Neurology, Perelman School of Medicine, University of Pennsylvania, Philadelphia, Pennsylvania, United States of America; 13 Department of Pediatrics, Division of Child Neurology, The Children's Hospital of Philadelphia, Philadelphia, Pennsylvania, United States of America; McMaster University, CANADA

## Abstract

We aimed to compare health care utilization of children with pediatric-onset multiple sclerosis to that of age, sex and geographically-matched children without multiple sclerosis. Using population-based administrative data from Ontario, Canada for the period 2003–2014, we applied a validated case definition to identify persons aged ≤18 years with multiple sclerosis. We identified up to 5 children without multiple sclerosis matched on sex, age, and region of residence. In each cohort, we determined annual rates of any hospitalization and physician services use. Using general linear models we compared utilization rates adjusting for age, sex, region, socioeconomic status and year. Subsequently, we limited the analysis to incident cases of multiple sclerosis and their matches, and compared rates of utilization in the year of multiple sclerosis diagnosis, and the three years thereafter. We identified 659 youth with multiple sclerosis (428 incident cases), and 3,294 matched controls. Two-thirds of both cohorts were female. After adjustment for sociodemographic factors and year, the multiple sclerosis cohort was more likely to be hospitalized than the matched cohort (odds ratio 15.2; 95%CI: 12.0, 19.1), and had higher rates of ambulatory physician visits (rate ratio 4.58; 95%CI: 4.26, 4.92). The odds of hospitalization (odds ratio 40.1; 95%CI: 27.1, 59.5) and physician visits (rate ratio 5.14; 95%CI: 4.63, 5.71) were markedly elevated in the year of MS diagnosis, declining thereafter but remaining elevated versus the matched cohort. Children with multiple sclerosis have substantially elevated rates of health care utilization as compared to matched children without multiple sclerosis, over calendar time and throughout the early disease course.

## Introduction

Multiple sclerosis (MS) is a disease of the central nervous system which most commonly presents in adulthood. Affected adults have higher rates of health care utilization as compared to age and sex-matched adults without MS,[1, 2] but utilization in this population has changed over time, most notably due to a decrease in hospitalization rates.[1, 3] In about 5% of individuals with MS, the initial presentation is in childhood or adolescence. However, relatively little is known about health care utilization in the pediatric MS population. Prior studies have involved small cohorts, or have not been population-based.[4, 5]

Several factors suggest that health care use may differ in the pediatric and adult MS populations. According to the Anderson Behavioral Model, health care utilization is driven by predisposing factors (e.g. age), enabling or impeding factors which influence access to services (e.g. transportation), and the need for services (symptoms and illness).[6] Thus health care utilization may differ in the pediatric and adult populations due to predisposing factors and differences in the need for services. For example, annualized relapse rates are higher in the pediatric MS population than in the adult MS population.[7] Recovery from physical impairments induced by relapses may be greater in the pediatric population, but cognitive and behavioral impairments develop early.[8, 9] Further, childrens’ health care is also influenced by parental factors, particularly the pattern of maternal health care use and presence of maternal depression.[10, 11] Therefore it is important to specifically evaluate health care use in children with MS. Information about health care utilization is necessary to understand the burden of pediatric MS on the health system, predict future needs, and to evaluate the cost-benefit of treatment strategies. Therefore, we aimed to compare health care utilization of children with pediatric-onset MS with that of children without MS using population-based administrative (health claims) data.

## Materials and methods

### Setting

We conducted this population-based, retrospective cohort study in Ontario, Canada where approximately 2.9 million persons aged ≤18 years reside. Health care is provided to all residents through a publicly funded program, and records of services used are captured in electronic databases. Access to specialty care is obtained by referrals from primary care or emergency care providers.

### Data sources

We accessed databases held at ICES regarding health services use for the period April 1, 2003-March 31, 2014. These population-based databases (data used) included the registered persons database (unique identifier, date of birth, sex, region of residence based on postal code, and dates of health insurance coverage), the hospital discharge abstract database (dates of admission and separation, discharge diagnoses recorded using 5-digit International Classification of Disease (ICD)-9 or ICD-10-CA codes, depending on the year), and the Ontario Health Insurance Plan database (physician services including date and type of service, and one physician-assigned diagnosis recorded using 3-digit ICD-9 code). We did not access prescription claims data as these are not population-based for persons <65 years old. These anonymized datasets were linked using unique encoded identifiers and analyzed at ICES.

We obtained ethics approval from the Research Ethics Boards at Sunnybrook Health Sciences Centre (Toronto, Canada), the Hospital for Sick Children (Toronto, Canada), Children’s Hospital of Eastern Ontario (Ottawa, Canada), McMaster Children’s Hospital (Hamilton, Canada), and the Children’s Hospital of London Health Sciences Centre (London, Canada). Administrative data access (via ICES) was authorized under section 45 of Ontario’s Personal Health Information Protection Act, which does not require individual consent.

### Administrative case definition of MS

We applied a validated administrative case definition for pediatric MS (sensitivity 89.2%, specificity 100%),[12] which required ≥3 hospital or physician claims for MS as identified using International Classification of Disease (ICD)-9 or ICD-10-CA diagnostic codes (340/G35) using all available years of data. For each individual meeting the case definition, we looked backward from the first MS claim to identify the earliest ICD-9/10-CA claim for demyelinating disease (323, 340, 341, 377.3, H46, G35, G36, G37), and classified this as the index date. Next, for each case, we identified up to 5 controls matched on sex, exact year of birth, and region of residence (based on the first three digits of the postal code). Each control was assigned the index date of its matched case. Controls did not have any hospital or physician claims for demyelinating disease.

### Health care utilization

The health care utilization outcomes of interest were the annual rates of all-cause hospitalizations and physician visits. For hospitalizations we focused on overnight hospitalizations. To avoid double-counting hospitalizations related to transfers between facilities for continued care, overnight hospitalizations beginning within ±1 day of another hospital discharge with the same primary or secondary diagnosis codes were considered part of the same hospitalization. For physician visits we examined outpatient (face-to-face ambulatory) physician services use overall, and stratified by specialty (general practice/pediatrics, neurology, ophthalmology, physiatry, psychiatry). The specialties chosen were those thought to be most often involved in the care of children with MS. Services provided by non-physician providers are not captured.

### Covariates

Covariates included age (continuous), sex (male as reference group), study year (2003–2006 [reference group], 2007–2010, 2011–2014), socioeconomic status (SES) in quintiles (lowest quintile of SES as reference group), and region (urban or rural [reference group]). We derived SES by linking the postal code of residence to Canadian census data in 2006. Urban regions included communities with populations ≥100,000 based on the 2006 census metropolitan areas or census agglomeration.

### Analysis

We summarized categorical variables using frequency (percent) and continuous variables using mean (standard deviation [SD]) or median (interquartile range [IQR]). We examined health care utilization in two ways. First, we focused on patterns of utilization over the time period from 2003–2014. In each calendar year, prevalent cases of MS were included as long as it was after their index date, and they were ≤18 years old. For each utilization outcome of interest we report the crude, age- and sex-standardized rates for each cohort, stratified by year and 95% confidence intervals (95%CI) based on a gamma distribution; rates were standardized to the 2006 Canadian census population. Confidence intervals for rate ratios (RR) were calculated by bootstrapping.

We compared the occurrence of any hospitalizations between the MS and matched cohorts using both unadjusted and covariate-adjusted (see above) regression models. We used binomial regression and included the log of person-year years as the model offset to account for differential follow-up time. To account for repeated hospitalizations by individuals we used generalized estimating equations using an exchangeable correlation matrix. We also compared the number of ambulatory physician visits between the MS and matched cohorts using the same general approach, except that we used negative binomial regression to account for over-dispersion. Here, we report odds ratios (OR) and RR and 95%CI for the associations observed.

Second, we examined health care utilization relative to time from the index date. For these analyses, we limited the study population to incident MS cases,[2] by requiring a ≥5 year period with no demyelinating disease claims before the index date.[12] To allow a three year follow-up period after the index date we limited the latest index year to 2011. Consistent with prior work in the adult MS population, we designated the 6-month periods on either side of the index date as the “year of diagnosis” (aka Year 0), and the subsequent 12-month periods as years 1 through 3.[2] We repeated analyses of health care utilization rates described above according to time from the index date (Year 0–3) instead of calendar time. We repeated our regression analyses as described above with the following changes: (i) we added time from the index date (0, 1, 2, 3); (ii) we added an interaction term between cohort*year. Index year was categorized as 2003–2006, and 2007–2011. All other covariates remained the same as for the primary analysis.

Statistical analyses used SAS V9.4 (SAS Institute Inc., Cary, NC).

### Data sharing statement

ICES is a named prescribed entity, under section 45(1) of the *Personal Health Information Protection Act (PHIPA)*, 2004 and enables health information custodians (HICs) to disclose personal health information (PHI) to ICES for the purposes of analysis, compiling statistical and evaluative information related to the management, evaluation, monitoring of the health care system, allocation of resources and helping in the planning of the system–without individual consent. A variety of measures are deployed to protect the personal health information entrusted to ICES and, under the Personal Health Information Protection Act, the underlying data are legally not allowed for public repository. ICES will only release aggregate analysis results. All statistical analyses will be conducted at ICES facilities. Only the aggregate level findings of our analysis will be reported publicly. The dataset from this study is held securely in coded form at ICES. While data sharing agreements prohibit ICES from making the dataset publicly available, access may be granted to coded data to those who meet pre-specified criteria for confidential access, available at www.ices.on.ca/DAS.

## Results

We identified 659 children and adolescents living with MS during the study period. Of these, all but one had five matches for a total of 3,294 controls; thus the total sample for this study included 3,953 youth. The cohorts were well-matched (Table 1). Two-thirds of the cohorts were female. Most participants lived in urban areas. Health care utilization in the year before the index date (first demyelinating disease claim) was higher in the MS cohort.

**Table 1:** Characteristics of the study populations.

Characteristic	Prevalent Period = 2003–2014			Incident Period = 2003–2011		
	Multiple Sclerosis	Matched Controls	P-value	Multiple Sclerosis	Matched Controls	P-value
N	659	3294		428	2139	
Female, n (%)	410 (62.2)	2050 (62.2)	0.99	272 (63.6)	1,360 (63.6)	0.99
Age at index date, mean (SD)	14.1 (4.5)	14.1 (4.5)	0.95	14.9 (3.0)	14.9 (4.0)	0.93
Duration of follow-up, mean (SD)	3.8 (3.8)	3.8 (3.8)	0.87	3.1 (3.0)	3.1 (3.0)	0.93
Urban region of residence, n (%)	596 (90.4)	2963 (90.0)	0.70	383 (89.5)	1902 (88.9)	0.73
Socioeconomic status, n (%)			0.16			0.082
Quintile 1 (lowest)	144 (21.9)	617 (18.7)		91 (21.3)	398 (18.6)	
Quintile 2	112 (17.0)	616 (18.7)		74 (17.3)	407 (19.0)	
Quintile 3	127 (19.3)	658 (20.0)		84 (19.6)	443 (20.7)	
Quintile 4	124 (18.8)	707 (21.5)		74 (17.3)	459 (21.5)	
Quintile 5 (highest)	152 (23.1)	696 (21.1)		105 (24.5)	432 (20.2)	
Health care utilization, n (%)						
Hospitalized in the year before the index date, n (%)	109 (16.5)	66 (2.0)	<0.001	63 (14.7)	40 (1.9)	<0.001
Number of physician visits in the year before the index date, median (IQR)	9 (5–16)	3 (1–6)	<0.001	9 (5–15)	3 (1–6)	<0.001

### Health care utilization among prevalent cases after the index date

#### All-cause hospitalizations

**Fig 1:**
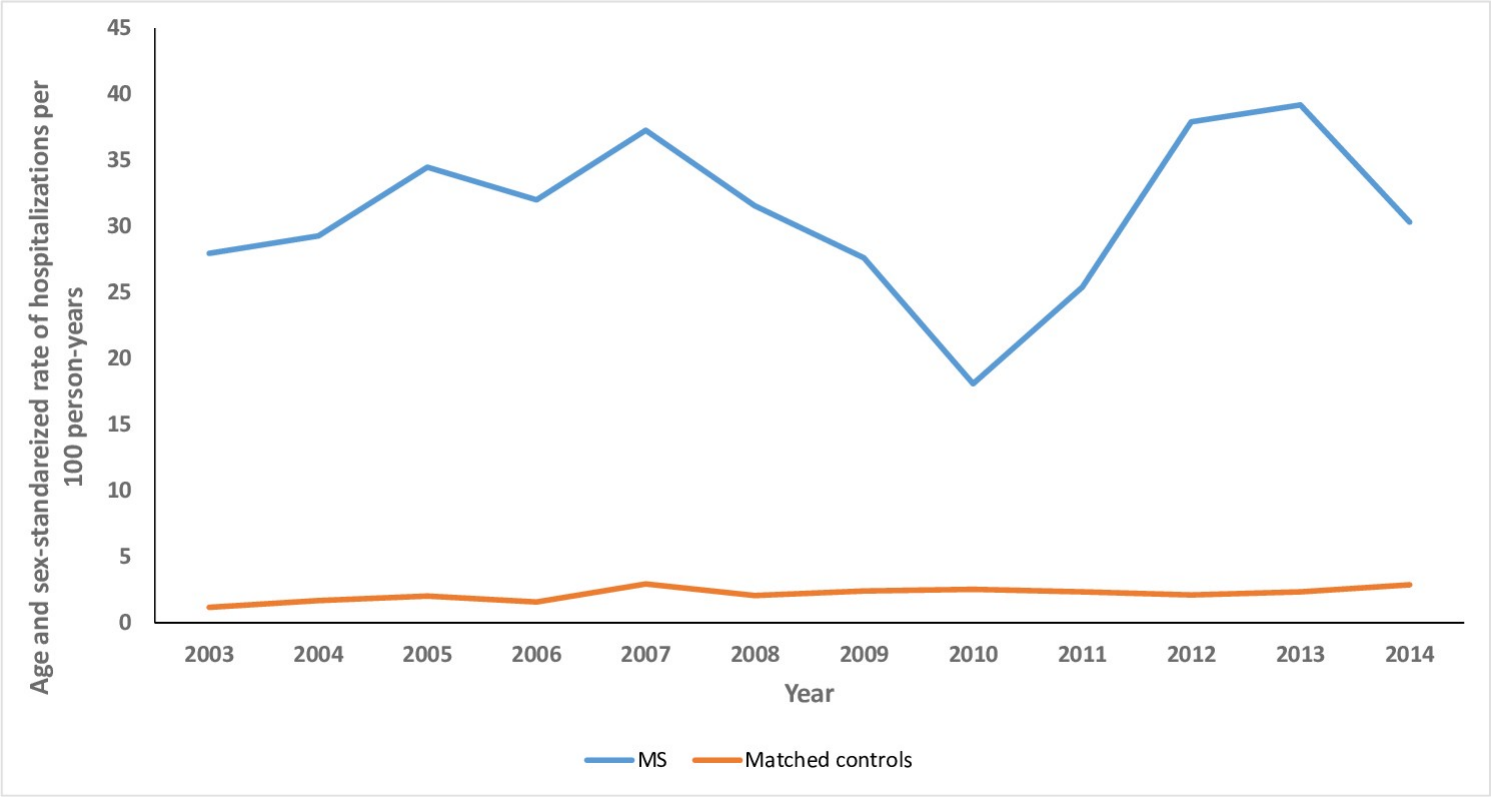
Age and sex-standardized rate of hospitalizations/100 person-years in the pediatric multiple sclerosis and matched cohorts.

The annual rate of hospitalizations was consistently higher in the MS cohort than in the matched cohort throughout the study period (Fig 1). In 2014, the MS cohort had a crude annual rate of hospitalizations of 34.1 per 100 person-years (95%CI: 25.5–44.8). After age and sex-standardization, the annual rate of hospitalizations was 30.3 per 100 person-years (95%CI: 21.6–41.4), 10-fold higher than in the matched cohort (crude rate 3.1 per 100 person-years [95%CI: 2.0–4.6]; standardized rate per 100 person-years 2.9 [95%CI: 1.7–4.4]).

After adjusting for age, sex, SES and region, the MS cohort was substantially more likely to be hospitalized than the matched cohort (OR 15.2; 95%CI: 12.0–19.1, Table 2). Older age was associated with an increased likelihood of hospitalization. Males and children of highest SES (versus lowest SES) had a reduced likelihood of hospitalization.

**Table 2:** Adjusted associations between multiple sclerosis and any hospitalizations, and rate of ambulatory physician visits.

	Any hospitalization	Ambulatory physician visits
Variable	Adjusted Odds Ratio^a^ (95% CI)	Adjusted Rate Ratio^b^ (95% CI)
*Population*		
Matched controls	1.0	1.0
MS	**15.2** **(12.0, 19.1)**	**4.58** **(4.26, 4.92)**
Age	**1.03** **(1.01, 1.06)**	**1.023** **(1.018, 1.028)**
*Sex*		
Female	1.0	1.0
Male	**0.69** **(0.55, 0.87)**	**0.75** **(0.71, 0.79)**
*Socioeconomic Status*		
Quintile 1 (lowest)	1.0	1.0
Quintile 2	0.85 (0.59, 1.22)	**0.90** **(0.82, 0.99)**
Quintile 3	0.75 (0.53, 1.06)	**0.89** **(0.81, 0.97)**
Quintile 4	0.74 (0.51, 1.06)	**0.99** **(0.91, 1.09)**
Quintile 5 (highest)	**0.51** **(0.35, 0.73)**	0.99 (0.91, 1.09)
*Region*		
Urban	1.0	1.0
Rural	0.77 (0.53, 1.13)	1.06 (0.99, 1.14)
*Year*		
2004–2006	1.0	1.0
2007–2010	0.92 (0.71, 1.21)	1.06 (0.99, 1.14)
2011–2014	1.14 (0.87, 1.48)	1.00 (0.94, 1.08)

a Binomial regression model with generalized estimating equations.

b- Negative binomial regression with generalized estimating equations. Bold indicates statistical significance

#### Physician visits

**Fig 2:**
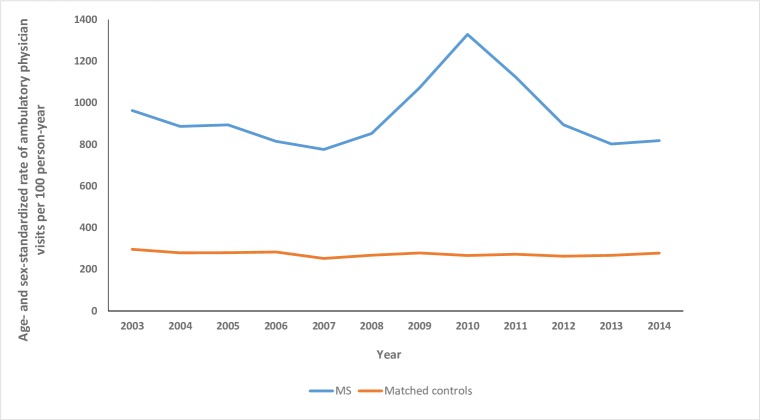
Age and sex-standardized rate of ambulatory physician visits/100 person-years in the pediatric multiple sclerosis and matched cohorts.

Over the study period, the crude annual number of ambulatory physician visits in the MS cohort ranged from 828.2 (95%CI: 783.9–874.2) to 1703.3 (95%CI: 1643.9–1764.4) per 100 person-years. After age and sex-standardization, the rate of physician visits was consistently ≥3-fold higher in the MS cohort than in the matched cohort (Fig 2). The standardized rates of ambulatory visits were higher in the MS cohort than in the matched cohort for all specialties examined (Table 3). In 2014, as compared to rates in the non-MS cohort, the annual rates of physician visits in the MS cohort were 2-fold higher for primary care (RR 2.27; 95%CI: 2.25–2.29), nearly 3-fold higher for psychiatry (RR 2.97; 95%CI: 2.74–3.11), 18-fold higher for ophthalmology (RR 18.5; 95%CI: 18.0–18.7), and 100-fold higher for neurology (RR 109.4; 95%CI: 107.2–111.2). Rates and RR for physical medicine are not shown in Table 3 because these were zero or nearly zero in the control cohort in several years. In 2014, the rate of visits to physical medicine was 1.95 per 100 person-years (95%CI: 0.63–4.55) in the MS cohort and 0 in the matched cohort.

**Table 3:** Age and sex-standardized rates of office visits per 100 person-years by physician specialty.

	Primary Care			Neurology			Psychiatry			Ophthalmology		
Year	MS (95%CI)	Matched Controls (95%CI)	RR (95%CI)	MS (95%CI)	Matched Controls (95%CI)	RR (95%CI)	MS (95%CI)	Matched Controls (95%CI)	RR (95%CI)	MS (95%CI)	Matched Controls (95%CI)	RR (95%CI)
2003	546.2 (505.0, 589.9)	253.8 (240.7, 267.9)	**2.15** **(2.12, 2.17)**	215.6 (189.1, 244.8)	0.25 (0.027, 0.96)	**852.2** **(813.4, 865.2)**	22.4 (15.1, 32.0)	5.56 (3.78, 7.89)	**4.02** **(3.59, 4.11)**	47.9 (36.0, 62.6)	5.19 (3.37, 7.64)	**9.24** **(8.84, 9.54)**
2004	519.3 (479.5, 561.5)	235.9 (223.2, 249.1)	**2.20** **(2.16, 2.22)**	209.3 (183.2, 238.1)	0.83 (0.33, 1.72)	**252.2** **(245.9, 255.4)**	28.1 (20.1, 38.2)	5.12 (3.36, 7.48)	**5.49** **(5.01, 5.59)**	36.0 (26.0, 48.8)	5.04 (3.25, 7.47)	**7.16** **(6.63, 7.35)**
2005	528.9 (491.1, 568.8)	236.9 (225.3, 249.0)	**2.23** **(2.19, 2.26)**	198.0 (174.7, 223.5)	1.36 (0.63, 2.57)	**145.3** **(142.5, 146.9)**	36.0 (26.5, 47.7)	3.98 (2.62, 5.80)	**9.04** **(7.96, 9.19)**	33.7 (24.7, 45.0)	3.54 (2.25, 5.30)	**9.54** **(8.92, 9.68)**
2006	440.4 (406.6, 476.3	237.9 (226.5, 249.6)	**1.85** **(1.82, 1.87)**	185.9 (164.1, 209.9)	0.73 (0.26, 1.63)	**253.3** **(242.6, 255.9)**	17.3 (11.7, 24.8)	4.87 (3.45, 6.67)	**3.56** **(3.19, 3.61)**	36.4 (27.3, 47.6)	3.50 (2.27, 5.17)	**10.4** **(9.89, 10.5)**
2007	428.2 (396.8, 462.4)	215.9 (205.2, 227.0)	**1.98** **(1.96, 2.00)**	198.7 (177.2, 222.0)	1.78 (0.95, 3.05)	**111.4** **(110.1, 112.1)**	16.8 (11.3, 24.0)	3.27 (2.14, 4.78)	**5.14** **(4.91, 5.25)**	38.4 (29.3, 49.4)	3.00 (1.87, 4.55)	**12.8** **(12.4, 12.9)**
2008	438.1 (405.5, 472.8)	227.6 (217.0, 238.7)	**1.92** **(1.90, 1.94)**	206.7 (184.9, 230.5)	2.15 (1.23, 3.50)	**96.0** **(94.4, 97.3)**	24.2 (17.3, 33.1)	6.54 (4.97, 8.44)	**3.71** **(3.54, 3.76)**	63.1 (51.4, 76.7)	2.56 (1.58, 3.92)	**24.6** **(23.9, 24.9)**
2009	762.8 (720.9, 804.5)	231.1 (220.7, 241.9)	**3.30** **(3.24, 3.35)**	140.9 (123.6, 159.9)	1.11 (0.52, 20.7)	**127.2** **(124.2, 128.1)**	17.7 (11.9, 25.3)	8.89 (7.18, 10.9)	**1.99** **(1.94, 2.04)**	54.3 (43.6, 67.0)	2.46 (1.55, 3.72)	**22.1** **(21.5, 22.4)**
2010	1037.9 (993.9, 1081.2)	221.0 (210.0, 231.4)	**4.69** **(4.63, 4.77)**	1.15 (1.00, 1.33)	1.99 (1.17, 3.15)	**58.1** **(57.3, 58.6)**	15.3 (9.61, 23.1)	8.48 (6.80, 10.4)	**1.81** **(1.72, 1.85)**	43.3 (33.6, 55.0)	2.56 (1.58, 3.92)	**16.9** **(16.4, 17.1)**
2011	747.8 (707.6, 789.7)	221.9 (211.7, 232.4)	**3.37** **(3.30, 3.41)**	178.2 (158.3, 199.9)	0.79 (0.35, 1.54)	**225.6** **(224.5, 227.6)**	24.4 (17.6, 33.1)	9.57 (7.75, 11.7)	**2.56** **(2.42, 2.60)**	53.2 (42.2, 66.4)	1.97 (1.19, 3.07)	**27.0** **(26.1, 27.4)**
2012	584.2 (544.5, 625.6)	212.9 (202.4, 223.9)	**2.74** **(2.71, 2.78)**	118.1 (101.0, 137.1)	1.16 (0.48, 2.34)	**101.9** **(99.1, 103.0)**	19.5 (12.9, 28.4)	3.79 (26.1, 5.31)	**5.17** **(4.99, 5.33)**	63.0 (51.0, 77.0)	2.35 (1.45, 3.61)	**26.8** **(26.1, 27.4)**
2013	515.5 (478.3, 554.8)	215.6 (204.8, 226.8)	**2.39** **(2.37, 2.42)**	110.7 (93.2, 130.5)	1.67 (0.92, 2.78)	**66.3** **(64.8, 66.9)**	15.5 (9.92, 23.2)	11.6 (9.33, 14.3)	**1.34** **(1.23, 1.36)**	49.6 (38.3, 63.1)	2.59 (1.58, 4.02)	**19.1** **(18.2, 19.4)**
2014	526.4 (487.5, 568.5)	231.8 (220.6, 243.5)	**2.27** **(2.25, 2.29)**	121.7 (103.2, 142.6)	1.11 (0.48, 2.19)	**109.4** **(107.2, 111.2)**	24.0 (15.4, 35.5)	8.08 (60.5, 10.6)	**2.97** **(2.74, 3.11)**	49.4 (38.6, 62.2)	2.67 (1.59, 4.22)	**18.5** **(18.0, 18.7)**

RR = Rate Ratio; 95%CI: 95% confidence interval; bold = statistical significance

After adjusting for age, sex, SES and region, the MS cohort had a 4.5-fold higher rate of ambulatory physician visits than the matched cohort (RR 4.58; 95%CI: 4.26–4.92) (Table 2). Older age was associated with an increased rate of physician visits. Males, and children of higher SES (versus lowest SES) had a lower rate of visits.

### Health care utilization according to time from diagnosis among incident cases

**Fig 3:**
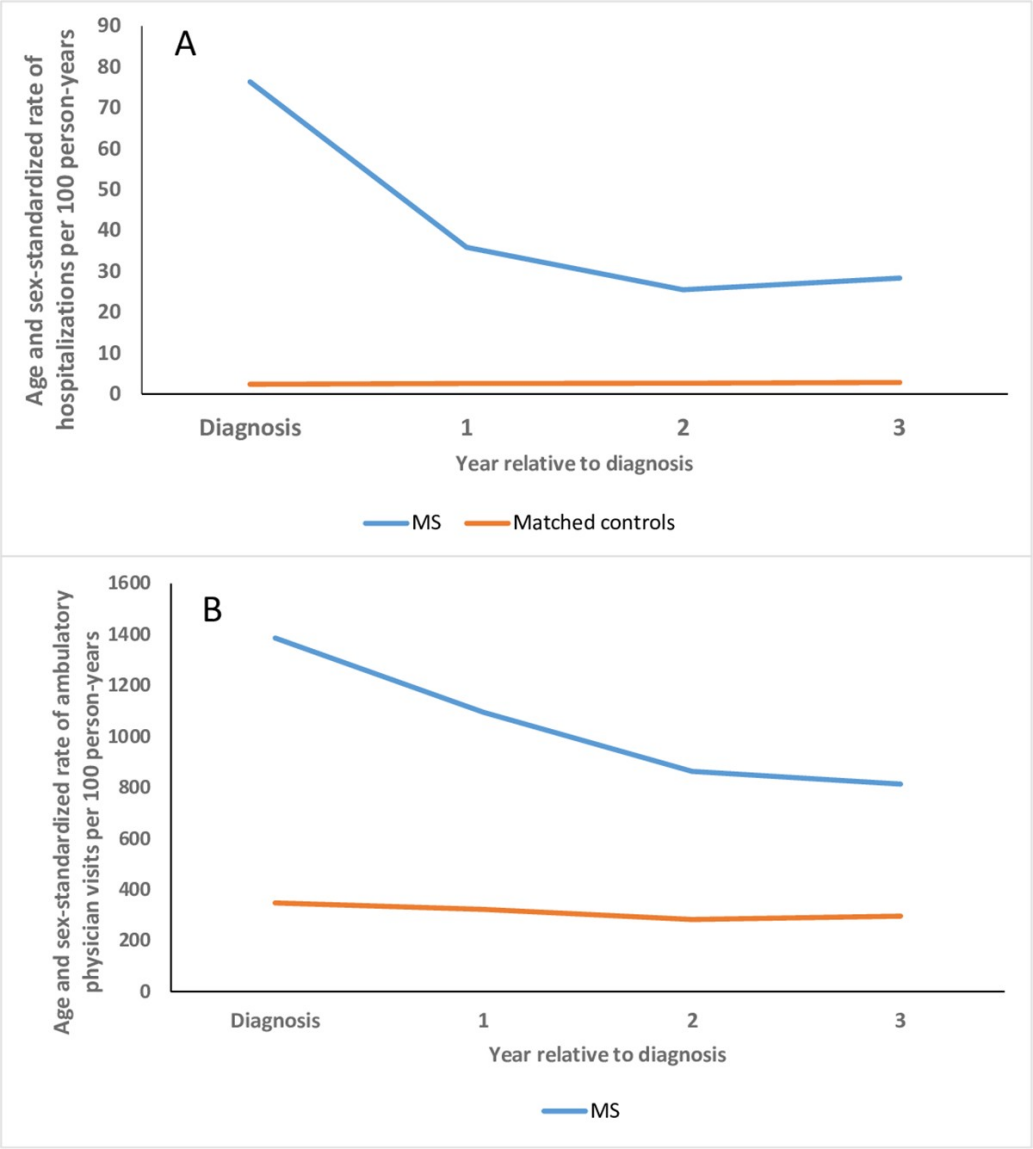
Rates of hospitalization (A) and ambulatory physician visits (B) according to time from diagnosis in the multiple sclerosis (MS) and matched cohorts.

Characteristics of the incident MS cases (n = 428) and their matched controls (n = 2,139) were similar to those of the prevalent cases (Table 1). In the “year of diagnosis”, after age and sex-standardization, the rate of hospitalization per person-year was >30-fold higher in the MS cohort than in the matched cohort (RR 32.3; 95%CI: 32.0–32.8). In the MS cohort, the rate of hospitalizations was substantially lower in the first, second and third years after diagnosis but remained elevated versus the matched cohort (Fig 3A). The overall pattern of findings was similar for ambulatory physician visits overall (Fig 3B) as for neurology and ophthalmology visits (Table 4). For primary care the relative rate of visits was stable by time since diagnosis. For psychiatry the rate of visits declined from the “year of diagnosis” to year 2, then increased again in year 3.

**Table 4:** Age and sex-standardized rates of office visits by physician specialty according to time from diagnosis in the multiple sclerosis (MS) and matched cohorts.

	Primary Care			Neurology			Psychiatry			Ophthalmology		
Year	MS (95%CI)	Matched Controls (95%CI)	RR (95%CI)	MS (95%CI)	Matched Controls (95%CI)	RR (95%CI)	MS (95%CI)	Matched Controls (95%CI)	RR (95%CI)	MS (95%CI)	Matched Controls (95%CI)	RR (95%CI)
Y0	883.4 (834.0, 934.9)	308.3 (292.6, 324.5)	**2.87** **(2.84, 2.89)**	236.1 (212.2, 261.9)	0.74 (0.32, 1.46)	**320.3** **(317.8, 323.7)**	20.7 (14.1, 29.3)	5.07 (3.78, 6.67)	**4.08** **(4.01, 4.20)**	102.4 (844.6, 122.8)	3.79 (2.32, 5.83)	**27.1** **(26.8, 27.4)**
Y1	710.4 (669.4, 753.2)	276.1 (262.0, 290.7)	**2.57** **(2.54, 2.59)**	182.4 (150.2, 206.9)	0.70 (0.28, 1.44)	**261.0** **(258.1, 263.0)**	21.8 (15.2, 30.2)	7.26 (5.84, 8.92)	**2.99** **(2.89, 3.07)**	52.7 (40.6, 67.4)	3.03 (1.80, 4.78)	**17.4** **(17.0, 17.7)**
Y2	571.5 (532.6, 612.4)	239.5 (227.2, 252.3)	**2.39** **(2.36, 2.41)**	124.7 (106.7, 144.9)	1.97 (1.17, 3.11)	**63.2** **(61.9, 64.2)**	17.1 (10.5, 26.4)	6.76 (5.51, 8.21)	**2.53** **(2.40, 2.63)**	43.6 (31.9, 58.1)	2.27 (1.19, 3.93)	**19.2** **(18.6, 19.5)**
Y3	529.9 (478.2, 585.7)	251.0 (234.4, 268.4)	**2.11** **(2.08, 2.15)**	120.9 (98.9, 146.4)	2.80 (1.04, 6.05)	**43.2** **(42.0, 43.9)**	19.8 (12.5, 29.7)	3.47 (2.53, 4.64)	**5.69** **(5.42, 5.97)**	33.8 (23.7, 46.9)	1.84 (1.09, 2.92)	**18.4** **(17.4, 18.9)**

Y = Year; RR = Rate Ratio; 95%CI: 95% confidence interval; bold = statistical significance

On multivariable regression analysis, the odds of any hospitalization in the MS cohort were markedly elevated as compared to the matched cohort in the “year of diagnosis” (OR 40.1; 95%CI: 27.1–59.5). The odds of hospitalization remained elevated thereafter although to a lesser degree (year 1: OR 6.75; 95%CI: 4.30–10.6; year 2: OR 5.96; 95%CI: 3.37–10.5; year 3: OR 7.25; 95%CI: 3.65–14.4). Region of residence was not associated with the odds of hospitalization (OR 0.98; 95%CI: 0.66–1.47) but older age was (OR 0.92; 95%CI: 0.90–0.95). Male sex was associated with reduced odds of hospitalization (OR 0.78; 95%CI: 0.60–1.01). The highest (versus lowest) level of SES was associated with reduced odds of hospitalization (OR 0.60; 95%CI: 0.41–0.89).

On multivariable regression analysis, physician visit rates in the MS cohort were elevated in the “year of diagnosis” (RR 5.42; 95%CI: 4.82–6.09) as compared to the matched cohort. The rates of physician visits in the MS cohort remained elevated in subsequent years although less (year 1: RR 4.08; 95%CI: 3.51–4.74; year 2: RR 3.01; 95%CI: 2.52–3.60; year 3: RR 2.86; 95%CI: 2.50–3.27). Older age was associated with an increased rate of physician visits (RR 1.02; 95%CI: 1.00–1.04). SES were not associated with the rates of physician visits (highest versus lowest quintile RR 0.96; 95%CI: 0.81–1.13). Male sex (RR 0.82; 95%CI: 0.71–0.95) and rural versus urban residence (RR 0.75; 95%CI: 0.63–0.90) were associated with reduced rates of use.

## Discussion

In this population-based cohort study, health care utilization was higher in the pediatric MS population than in a matched population without MS, after accounting for predisposing (age, sex) and enabling factors (region and SES). Male youth had a lower likelihood of hospitalization and lower rates of physician visits than female youth. Older age was associated with an increased likelihood of hospitalization and higher rates of physician visits. In the three years after diagnosis, rural residence was associated with lower health care utilization, suggesting that access to care factors such as distance and transportation costs influence use. Such disparities could be differentially affected by severity of MS, but we lacked the clinical information to evaluate this. The increased use of ambulatory physician services by the MS cohort extended to primary care and specialty care. Consistent with the increased risk of mental health and behavioral concerns in MS,[13] and frequent involvement of the eyes (optic neuritis), and potentially physically disabling relapses, visits to psychiatry, ophthalmology and physiatry were more frequent. This highlights the importance of multidisciplinary care in pediatric MS. However, our findings do not indicate whether the needs of children with MS are being fully met.

Rates of health care utilization in the prevalent MS and matched cohorts were largely consistent over calendar time, including hospitalizations, although we observed a transient drop in hospitalizations in 2010 which was coupled with an increase in ambulatory physician visits. Previously, we reported that the incidence of pediatric-onset MS increased in Ontario in 2009, then dropped transiently before stabilizing, which was mirrored in the adult-onset MS population.[14] The stability of hospital admission rates over time differs from the adult-onset MS population in which hospitalization rates have decreased substantially over the last twenty years,[1, 3] exceeding declines in the general population.[2] This may reflect more frequent admissions for relapses and treatment in the pediatric population, which is now uncommon in the adult MS population.[2]

Among incident MS cases, health care utilization was markedly elevated in the “year of diagnosis”, that is, in the 6-month period before and after the index date, particularly for hospitalizations, reflecting that most children with incident demyelinating events in Canada are hospitalized.[15] The rate of health care utilization declined following diagnosis but remained elevated thereafter, consistent with findings in the adult MS population in Manitoba.[2]

Notably, health care use was elevated in the 12-month period before the index date. In the adult MS population, the rates of hospitalizations, physician visits, and prescription use increase between one and five years before the first demyelinating disease claim.[16] This has been interpreted as evidence of a prodromal period, consistent with findings that high school performance was worse in individuals with MS than in controls on average 13 years before the onset of MS symptoms.[17] Future studies should further evaluate the possibility of a prodromal period in pediatric-onset MS, as this is important for determining the etiologically-relevant time period for exposure to potential risk factors.

Little comparable work exists regarding health care utilization in the pediatric MS population, and it comes from the American health care system, which includes a mixture of public and private sources of care, unlike the public, universally funded Ontario system. In an American study that used the Pediatric Health Information System database the annual rate of hospital admissions among persons <19 years old rose 1.5-fold in a decade, from 34.7 per 1,000 population in 2003 to 53.2 in 2013. However, the prevalence of pediatric MS in the surrounding regions was unknown, making it uncertain whether this reflects an increase in the number of children with MS needing care, or changes in care delivery.[5] A study of 57 children with MS from Utah found that the mean number of hospitalizations was 1.2, and of outpatient visits was 22.7 over a 10-year period.[4] Neither study reported comparative findings for children without MS. In the broader population, American children with a chronic health condition requiring care and limiting daily function had four times more hospitalizations and twice as many physician visits as children without such conditions in the year 2000.[18]

This study has some limitations. We lacked population-based data regarding outpatient prescription medications, and could not determine whether, or how, medication use differed between the MS and non-MS populations. We employed an area-based measure of SES rather than an individual-level measure; however, area-based measures of SES are associated with health status,[19] and produce similar findings as individual-level measures when the geographic regions used are small.[20] Pediatric specialists at some tertiary care centres participate in alternative funding plans, and shadow billing may not have fully captured use of their services. This would underestimate health services use, and tend to bias RR comparing utilization in the MS and matched cohorts toward the null, suggesting that the effect of pediatric MS on health services may be greater than we have reported. The data sources used also do not provide information regarding the use of non-physician providers such as physiotherapists or psychologists. We did not investigate the use of diagnostic testing, including laboratory tests and imaging. We lacked clinical measures of health, and future studies should examine what specific clinical features lead to increased health care use in the pediatric MS population. The study was limited to one Canadian province; the findings may differ in other jurisdictions as care varies widely for children with complex health care needs.[21] Strengths of this study include the population-based design, use of a validated case definition to identify youth with MS, use of a matched cohort from the general population for comparison, and a 10-year study period.

The burden of health care use in the pediatric MS population is high, and substantially exceeds health care use in an age, sex and geographically-matched pediatric population without MS. Health care use is particularly high during the year of diagnosis, and remains elevated thereafter.

## References

[1] Marrie RA, Elliott L, Marriott J, Cossoy M, Blanchard J, Tennakoon A, et al Dramatically changing rates and reasons for hospitalization in multiple sclerosis. Neurology. 2014;83(10):929–37. 10.1212/WNL.0000000000000753 25085638 PMC4153848

[2] Marrie RA, Yu N, Wei Y, Elliott L, Blanchard J. High rates of physician services utilization at least five years before multiple sclerosis diagnosis. Multiple Sclerosis Journal. 2013;19(8):1113–9. Epub 2012/12/25. 1352458512471877 [pii] 10.1177/1352458512471877 . 23263898

[3] Evans C, Kingwell E, Zhu F, Oger J, Zhao Y, Tremlett H. Hospital admissions and MS: temporal trends and patient characteristics. Am J Manag Care. 2012;18(11):735–42. Epub 2012/12/04. 80713 [pii]. . 23198715

[4] Wright MA, Korgenski EK, Bardsley T, Bonkowsky JL, Candee MS. Comprehensive population-based determination of pediatric multiple sclerosis health care costs. Neurol Neuroimmunol Neuroinflamm. 2017;4(1):e314 Epub 2016/12/27. 10.1212/NXI.0000000000000314 28018945 PMC5173349

[5] Lavery AM, Banwell BL, Liu G, Waldman AT. Hospital admission rates for pediatric multiple sclerosis in the United States using the Pediatric Health Information System (PHIS). Mult Scler Relat Disord. 2016;9:5–10. Epub 2016/09/21. 10.1016/j.msard.2016.05.018 27645335 PMC5609002

[6] Andersen RM. National Health Surveys and the Behavioral Model of Health Services Use. Med Care. 2008;46(7):647–53. 10.1097/MLR.0b013e31817a835d 18580382

[7] Gorman MP, Healy BC, Polgar-Turcsanyi M, Chitnis T. Increased relapse rate in pediatric-onset compared with adult-onset multiple sclerosis. Arch Neurol. 2009;66(1):54–9. 10.1001/archneurol.2008.505 19139299

[8] Amato MP, Goretti B, Ghezzi A, Lori S, Zipoli V, Portaccio E, et al Cognitive and psychosocial features of childhood and juvenile MS. Neurology. 2008;70(20):1891–7. 10.1212/01.wnl.0000312276.23177.fa 18474844

[9] Amato MP, Krupp LB, Charvet LE, Penner I, Till C. Pediatric multiple sclerosis. Cognition and mood. 2016;87(9 Supplement 2):S82–S7. 10.1212/wnl.0000000000002883 27572867

[10] Riley AW, Finney JW, Mellits ED, Starfield B, Kidwell S, Quaskey S, et al Determinants of children's health care use: an investigation of psychosocial factors. Med Care. 1993;31(9):767–83. Epub 1993/09/01. . 8366679 10.1097/00005650-199309000-00002

[11] Minkovitz CS, Strobino D, Scharfstein D, Hou W, Miller T, Mistry KB, et al Maternal Depressive Symptoms and Children's Receipt of Health Care in the First 3 Years of Life. Pediatrics. 2005;115(2):306–14. 10.1542/peds.2004-0341 15687437

[12] Marrie RA, O'Mahony J, Maxwell C, Ling V, Yeh EA, Arnold DL, et al Incidence and prevalence of MS in children: a population-based study in Ontario, Canada. Neurology. 2018;91(17):e1579–e90. 10.1212/WNL.0000000000006395 30258022

[13] Goretti B, Ghezzi A, Portaccio E, Lori S, Zipoli V, Razzolini L, et al Psychosocial issue in children and adolescents with multiple sclerosis. Neurol Sci. 2010;31(4):467–70. Epub 2010/05/11. 10.1007/s10072-010-0281-x . 20454820

[14] Rotstein DL, Chen H, Wilton AS, Kwong JC, Marrie RA, Gozdyra P, et al Temporal trends in multiple sclerosis prevalence and incidence in a large population. Neurology. 2018;90(16):e1316–e23. 29549225 10.1212/WNL.0000000000005331

[15] O'Mahony J, Marrie RA, Laporte A, Yeh EA, Bar-Or A, Phan C, et al Recovery From Central Nervous System Acute Demyelination in Children. Pediatrics. 2015;136(1):e115–23. Epub 2015/06/03. 10.1542/peds.2015-0028 . 26034241

[16] Wijnands JMA, Kingwell E, Zhu F, Zhao Y, Hogg T, Stadnyk K, et al Health-care use before a first demyelinating event suggestive of a multiple sclerosis prodrome: a matched cohort study. The Lancet Neurology. 2017;16(6):445–51. Epub 2017/04/25. 10.1016/S1474-4422(17)30076-5 . 28434855

[17] Sinay V, Perez Akly M, Zanga G, Ciardi C, Racosta JM. School performance as a marker of cognitive decline prior to diagnosis of multiple sclerosis. Multiple Sclerosis Journal. 2015;21(7):945–52. 10.1177/1352458514554054 . 25344372

[18] Newacheck PW, Kim SE. A national profile of health care utilization and expenditures for children with special health care needs. Archives of Pediatrics & Adolescent Medicine. 2005;159(1):10–7. 10.1001/archpedi.159.1.10 15630052

[19] Krieger N. Overcoming the absence of socioeconomic data in medical records: Validation and application of a census-based methodology. American Journal of Public Health. 1992;82:703–10. 10.2105/ajph.82.5.703 1566949 PMC1694121

[20] Mustard CA, Derksen S, Berthelot J-M, Wolfson M. Assessing ecologic proxies for household income: a comparison of household and neighbourhood level income measures in the study of population health status. Health & Place. 1999;5(2):157–71. 10.1016/S1353-8292(99)00008-8. 10670997

[21] Ralston SL, Harrison W, Wasserman J, Goodman DC. Hospital Variation in Health Care Utilization by Children With Medical Complexity. Pediatrics. 2015;136(5):860–7. 10.1542/peds.2014-3920 26438701

